# Amorphous computing: examples, mathematics and theory

**DOI:** 10.1007/s11047-013-9370-0

**Published:** 2013-05-18

**Authors:** W. Richard Stark

**Affiliations:** Department of Mathematics & Statistics, University of South Florida, Tampa, FL 33620-5700 USA

**Keywords:** Amorphous computing, Computation theory, Distributed processes, Asynchronous, Probability measure

## Abstract

The cellular automata model was described by John von Neumann and his friends in the 1950s as a representation of information processing in multicellular tissue. With crystalline arrays of cells and synchronous activity, it missed the mark (Stark and Hughes, BioSystems 55:107–117, 2000). Recently, amorphous computing, a valid model for morphogenesis in multicellular information processing, has begun to fill the void. Through simple examples and elementary mathematics, this paper begins a computation theory for this important new direction.

## Introduction

Multicellular information processing is the computational basis for morphogenesis in living organisms. How can a process compute a pattern while working in a multicellular medium which is lacking a rigidly defined architecture for sharing information? The process is distributed over a network of similar cells, growing and dividing at different speeds, and reacting to different sets of neighbors. Chaotic irregularities in neighborhood structure, computational speed and in cell-to-cell communication seem to fly in the face of the strict order and control usually expected of computational processes. The creation of order in an asynchronous, randomly structured network of cells is the mystery of amorphous computing.

This paper presents a mathematical framework for amorphous processes which gracefully supports routine calculations, proofs of theorems and formal computation theory. First, the model is defined. Then more than a dozen processes are described. Their behavior is explored mathematically and by careful proofs of basic theorems. Useful mathematical structures (e.g., the state-transition graph, computations as Markov chains) develop out of applications of the model. The examples are seen to illustrate important programming styles. An algebraic approach to complexity is demonstrated through detailed calculations. The last half of the paper begins with proofs of non-computability results and an excursion into classical recursion theory. Finally, I conclude with a methodical construction of TearS—a complex dynamic amorphous process.

The wonderful possibility of extending computation theory to distributed systems such as these came to me in conversations with Professor John McCarthy (1978, Stanford University, Palo Alto, CA). Subsequently, I have received significant help in developing and publishing these ideas from Dr Leon Kotin (Fort Monmouth, NJ), Dr Edmund Grant (Tampa, FL), two very helpful referees and the editor of this journal. I sincerely thank all six of you.

… organisms can be viewed as made up of parts which, to an extent, are independent elementary units. … [A major problem] consists of how these elements are organized into a whole …” (John von Neumann 1948)

[A] mathematical model of a growing embryo [is] described … [it] will be a simplification and an idealization, … cells are geometrical points … One proceeds as with a physical theory and defines ‘the [global] state of the system. (Alan Turing 1952)

An amorphous computing medium is a system of irregularly placed, asynchronous, locally interacting computing elements. I have demonstrated that amorphous media can … generate highly complex pre-specified patterns. … [I]nspired by a botanical metaphor based on growing points and tropisms, … a growing point propagates through the medium. (Daniel Coore 1999)

## The model

A fixed finite state automata *A* is used to characterize the computational behavior of each cell in a multicellular network. The initial and accepting states are not used, so the automaton is represented by

$$ A = (Q,Q^+,\alpha,input) $$

where *Q* is the set of cell-values, *Q* ^+^ is the set of input values (describing multisets of values of neighboring cells), *α* is the value transition function (or relation in the non-deterministic case)

$$ \alpha:Q\times Q^+ \rightarrow Q $$

giving an active cell’s next value, and *input* is a function (or relation) which reduces the values of neighbors to a value in *Q* ^+^.

**Fig. 1:**
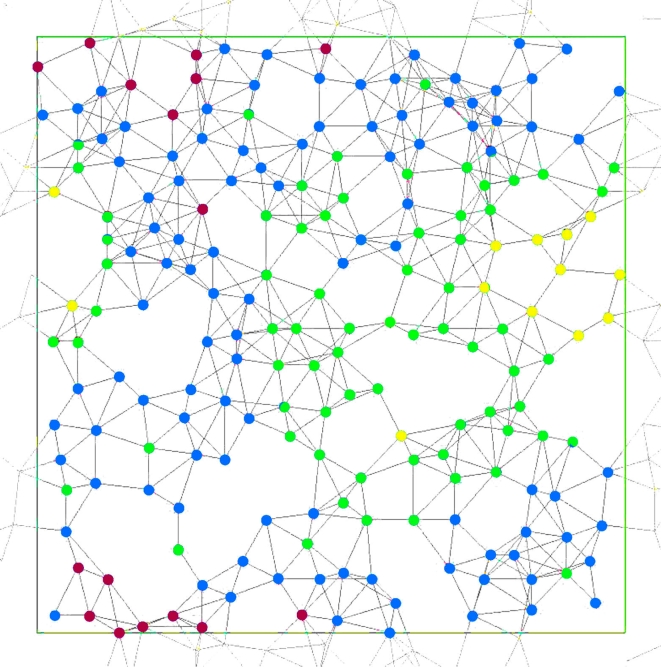
A network (*C*, *E*) of 200 cells on a torus for HearT (Sect. 7)

The network consists of a set *C* of cells connected by a set $E\subseteq C^2$ of edges. (*C*, *E*) is assumed to be a finite simple graph—i.e., edges are undirected (*cd* ∈ *E* if *dc* ∈ *E*), there are no self-edges ($cc \notin E$), and there are no multiple edges. Edges represent communication (with neighbors) (Fig. 1). Using *E* ambiguously, the set of cells neighboring a given cell *c* is denoted *E*(*c*) = {*d* | *cd* ∈ *E*}, and when *c* is included *E* ^+^(*c*) = *E*(*c*) ∪ {*c*}. The cell-values seen in *E*(*c*) give us a multiset

$$ M = \{q\in Q\ |\ q\; \hbox{is the cell-value of some}\;d\in E(c)\} $$

which characterizes *c*’s environment. In the theory (*C*, *E*) is a variable. The *input* function reduces multisets *M* to

$$ input(M) \in Q^+. $$

*Q* ^+^ is a finite set of values describing multisets of *Q*, so it may be convenient to include *Q* in *Q* ^+^. The cells of *E*(*c*) are anonymous (i.e., they have no addresses and so cannot be distinguished) so the various assignments giving for example *M* = {1, 0, 0} are not distinguished by *input*.

The term *state* is reserved for networks. A function *s*:  *C*→ *Q* indicating values *s*(*c*) ∈ *Q* for cells *c* is a (network) *state*. The multiset *M* of neighboring values at *c* is now

$$ s[E(c)]. $$

But *c* sees only *input*(*s*[*E*(*c*)]) ∈ *Q* ^+^. The set of all states is a set exponent

$$ Q^C $$

—a standard notation motivated by

$$||Q||^{||C||}=||\{s\ |\ s:C\rightarrow Q\}|| = ||Q^C||. $$

$(Q^C)^\mathbb{N},$ is the set of all infinite sequences of states.

An automaton *A* describes how each cell changes its value. Given a state *s*, an active cell *c* will change its value from *s*(*c*) to *α*(*s*(*c*), *input*(*s*[*E*(*c*)])). Communication is based on *c* *reading* information from its environment, rather than neighbors sending messages to *c*, so there will be no input buffers and no overflow problems.

Cell-activity in amorphous computing is asynchronous. Specifically, a state change is determined by the activity of a random set $\sigma\subseteq C$ of cells. Given *s* and *σ* the next state *s*′ is

$$ s^{\prime}(c) = \left\{\begin{array}{ll} \alpha(s(c),input(s[E(c)])) & \hbox{if}\; c \in \sigma \\ s(c) & \hbox{otherwise.} \end{array}\right. $$

This step is denoted *s* ⇒_*σ*_ *s*′. Write *s* ⇒ *s*′ when the transition is possible for some *σ*. Usually (if *α* and *input* are functions) non-determinism is restricted to the random choice of *σ*, and so *s* ⇒_*σ*_ *s*′ and *s*  ⇒_*σ*_ *s*′′ implies *s*′ = *s*′′.

A schedule $\sigma_0, \ldots,\sigma_n, \ldots$ of cell activity from an initial state *s*_0_ determines a *computation*

$$ s_0 \Rightarrow_{\sigma_0} \ldots s_n \Rightarrow_{\sigma_n}s_{n+1} \ldots. $$

Computations may be visualized as infinite paths through the tree of all finite sequences of states ordered by sequence-extension. If at some point *s*_*n*_  ⇒_*σ*_ *s*_*n*_ for all *σ* (equivalently *s*_*n*_ ⇒_*C*_ *s*_*n*_) then *s*_*n*_ is a *halting state* and the computation is said to *halt*. Whenever *s*(*c*) ≠ *s*′(*c*) for *s* ⇒_*c*_ *s*′, the cell *c* is said to be *unstable* in *s*.

The measurement of behavior is based on the probability of activity *P*_*a*_(*σ*) and the initial-state probability *P*_*i*_(*s*). Often *P*_*a*_(*σ*) = *δ* ^||*σ*||^ · (1 − *δ*)^||*C*−*σ*||^ where *δ* is the probability of a single cell being active and *P*_*i*_(*s*) = ||*Q*||^−||*C*||^. The probability of a computation step *s* ⇒ *s*′ is

$$ P_s(s,s^{\prime}) = \Upsigma_{\rho \subseteq \sigma\subseteq(\rho\cup\zeta)} P_a(\sigma)=\delta^{||\rho||}\cdot(1-\delta)^{||C-(\rho\cup\zeta)||} $$

where ζ = {*d* ∈ *C* | *s* ⇒_*d*_ *s*} is the set of cells *stable* in *s* and *ρ* = {*c* | *s*(*c*) ≠ *s*′(*c*)} the set of cells that must change their value. *P*_*s*_(*s*,*s*′) = 0 if not *s* ⇒ *s*′. For every $s, 1=\Upsigma_{t\in Q^C}P_s(s,t).$ The probability of a finite sequence being generated as a computation is

$$ P_c\{\langle\rangle\} = 1, \ P_c\{\langle s_0\rangle\} = P_i(s_0),\; \hbox{and}\; P_c\{\langle s_0, \ldots, s_m,s_{m+1}\rangle\} = P_c\{\langle s_0,\ldots s_m \rangle\}\cdot P_s(s_m,s_{m+1}). $$

For example *P*_*c*_{〈*s*_0_, *s*_0_〉} = *P*_*i*_(*s*_0_) · *P*_*s*_(*s*_0_, *s*_0_) = ||*Q*||^−||*C*||^ · (1 − *δ*)^*C*-ζ^, where ρ is empty and (*C* − ζ) is the set of cells not stable in *s*_0_ (and so not active).

Finally the probability measure μ on the set $(Q^C)^\mathbb{N}$ of all infinite sequences ${\overline s}$ of states begins on the basic open sets ${\mathcal O}^{\langle s_0, \ldots s_m\rangle}$ of infinite sequences extending 〈*s*_0_,… *s*_*m*_〉

$$ \mu({\mathcal O}^{\langle s_0,\ldots, s_m\rangle})= {{\mathcal{P}}}_c\{\langle s_0,\ldots, s_m\rangle\}, $$

and continues on to the Borel sets using Boolean operations and limits. For example, in a few minutes one can work from this definition to the measure of ${\mathcal C}$, the set of all computations, to get $\mu({\mathcal C})=1$ and so the infinite set $(Q^C)^\mathbb{N}-{\mathcal C}$ of infinite sequences which are not computations has measure 0. $(Q^C)^\mathbb{N}$ is an infinite set as large as the set of real numbers ℜ.

The analysis presented here sees behavior in the context of the set of all computations. The probability of success or failure is the μ-measure of corresponding sets of computations. 1 The potential decisions, which step-by-step generate branches filling out these sets of computations, should be viewed as thermodynamic events. In many ways, the set of $2^{\aleph_0}$ potential computations is analogous to the decimals of [0,1].

Finally, there are issues of permissible information. If we are to study amorphous computing then the introduction of information that is not computed is as forbidden to us as sneezing into a petri dish would be to a microbiologist. Thus, synchronous activity, serial activity and waves of activity are special cases falling outside of the ideal. The *ideal* I am speaking of is mathematical (the basis of mathematical tractability), it is not necessarily what we see in Nature.

Homogeneous programming has every cell using the same program—this is to avoid hiding information in the program assignment. But the cell program can have irrevocable branches which lead to different cells committed to different subprograms; this is acceptable because the pattern is computed from a random initial state in a uniform way.

These concerns lead to processes that I call *absolutely amorphous*. Absolutely amorphous processes are given the information in (*Q*, *Q* ^+^, *α*, *input*) only. They are important to the theory, but are not common in Nature. Daniel Coore’s model (2005) is amorphous but not absolutely amorphous. It allows some structure outside of *α* and *input*—e.g., non-random initialization of cells, cells with individual clocks, 2 and wave-like cell activity. After the non-computability result in Sect. 9, models that are not so absolutely amorphous take center stage.

Each (*C*, *E*) represents a particular architecture for sharing information. In order to study *amorphous* processes, (*C*, *E*) must vary freely as the general architecture-free theory evolves. Presently, (*C*, *E*) does not vary *while* a process is running; except in TearS, which is dynamic in that cells may die, new cells may be added and cells may move.

The investigations presented here build on this model—demonstrating its generality in examples, its tractability in calculations and proofs, and its power as a foundation for a computation theory. Except where credit is given, the analysis, theorems and proofs presented here are the work of the author.

## 2PartitioN

Imagine programming a living cell. Colonies of your cells eventually develop links 3 allowing neighboring cells to exchange information. You hope that the cell–cell communication will lead to a global behavior for the colony—even though the architecture and the speed with which cells execute their program is not under your control. Nevertheless, using only your ability to program a cell, you hope to orchestrate the behavior of the colony.

The first programming experiment is named 2PartitioN. In it, cells are finite-state automata with values $Q=\{0,1\}$ and with the ability to read the collective values of neighbors. According to the program, an active cell will change its value if it has a neighbor with the same value. In other words, if an active cell *c* with value *s*(*c*) has a neighbor *d* with *s*(*c*) = *s*(*d*), then it will change its value to 1 − *s*(*c*).

2PartitioN is defined by *Q* = {0, 1}, *Q* ^+^ = *Q* ∪ {01}, 

$$ \alpha(value,input) = \left\{\begin{array}{ll}1-value & \hbox{if}\; input=value \\& \hbox{or}\; input = 01 \\value & \hbox{otherwise,} \end{array}\right. $$

and for every multi-set *M* of neighboring cell-values

$$ input(M) = \left\{\begin{array}{ll} 0 & \hbox{if}\; M \; \hbox{contains only } 0 \hbox{s}, \\ 1  & \hbox{if}\; M \; \hbox{contains only } 1 \hbox{s}, \\ 01  & \hbox{otherwise.} \end{array}\right. $$

On *C*5, a ring of five cells, 2PartitioN never halts because a circuit of odd length cannot avoid a 00 or 11 edge. A halting state exists if and only if the colony’s network is bipartite. 4 i.e., our process can find a partition. This is true for every finite network. Thus, the network is a free variable, as it must be in amorphous computing, and 2PartitioN is a general program for determining bipartiteness. The input is the network.

Given $X\subseteq C$ the external *boundary*, ∂*X*, of *X*, is defined

$$ \partial X = \{b\in C\ |\ b\not\in X, bc\in E\;\hbox{for some}\;c\in X\}. $$

### **Theorem 1**

**Halting is Possible for 2PartitioN** *For bipartite* (*C*, *E*), *halting states exist and every* $s_0\Rightarrow_{\sigma_0}\ldots\Rightarrow_{\sigma_{m\text{-1}}}s_m$ *has an extension*

$$ s_0\Rightarrow_{\sigma_0} \ldots s_m\Rightarrow_{\sigma_m}\ldots\Rightarrow_{\sigma_{m+N\text{-1}}}s_{m+N}, (N\le||C||), $$

*with* *s*_*m*+*N*_ *halting. If* (*C*, *E*) *is not bipartite, then halting is impossible*. 5

### *Proof*

Assume that (*C*, *E*) is bipartite. For *s*_*m*_, let $X_m\subseteq C$ be a maximal connected set over which *cd*  ∈ (*E*∩ *X*_*m*_^2^) implies *s*_*m*_(*c*) ≠ *s*_*m*_(*d*), then

$$ s_m\Rightarrow_{\partial X_m}s_{m+1} $$

defines *s*_*m*+1_. If *s*_*m*_ is not halting, then *X*_*m*_ ≠ *C* and ∂*X*_*m*_ is a non-void set of unstable cells, and *s*_*m*+1_(*d*) = 1 − *s*_*m*_(*d*) = 1 − *s*_*m*_(*c*) = 1 − *s*_*m*+1_(*c*) for every *d* ∈ ∂*X*_*m*_. Choose *X*_*m*+1_ to be a maximal connected extension of *X*_*m*_ ∪ ∂*X*_*m*_ over which *s*_*m*+1_(*c*) ≠ *s*_*m*+1_(*d*)

$$ (X_m\cup \partial X_m)\subseteq X_{m+1}. $$

Continue with $s_{m+1} \Rightarrow_{{\partial X}_{m+1}} \; s_{m+2}$ etc. Since $X_m\subset X_{m+1}\subset \cdots \subseteq C$ the process reaches *C* before *N* = ||*C*|| steps. A fixed-point *X*_*m*_ = *X*_*m*+1_ implies that $\partial X_m\subseteq X_{m+1}-X_m$ is empty, and so *X*_*m*_ = *C*. At this point *s*_*m*+*N*_ is halting. This computation is just one of infinitely many. □

Another approach to proving such theorems begins with a halting state *r*. Then set *X*_*m*_ to be the maximal set (not necessarily connected) of cells *c* for which *s*_*m*_(*c*) = *r*(*c*). If *s*_*m*_ is not halting, then ∂*X*_*m*_ is non-void and we may continue as above.

### **Theorem 2**

**Non-Halting States are** ⇒-**Connected** *Given any* (*C*, *E*) *and any pair* *s*_0_, *t* *of states, with* *s*_0_ *non-halting, a computation* $s_0\Rightarrow\ldots\Rightarrow t$ *from* *s*_0_ *to* *t* *exists*.

### *Proof*

W.l.o.g, assume *cd* ∈ *E* such that *s*_0_(*c*) = *s*_0_(*d*). Let *X*_0_ be a maximal connected subset of *s*_0_^−1^(0) = {*c*|*s*_0_(*c*) = 0}, then $s_0 \Rightarrow_{{X}_{0}}\,s_1.$ If *X*_0_ has more than one cell, then every cell in *X*_0_ is unstable, so $X_0\subseteq s_1^{-1}(1).$ For odd *j*, let *X*_*j*_ be the maximal connected subset of *s*_*j*_^−1^(1) which extends *X*_*j*-1_; for even *j*, let *X*_*j*_ be the maximal connected subset of *s*_*j*_^−1^(0) which extends *X*_*j*{-1_. Then $s_j \Rightarrow_{{X}_{j}}\,s_{j+1}.$ When *s*_*k*_^−1^(0) = *C*, we set *X*_*k*_ = *t* ^−1^(1) and get $s_k \Rightarrow_{{X}_{k}}\,t.$ □

### **Theorem 3**

**2PartitioN Halts on Bipartite Networks** *Given a bipartite* (*C*, *E*) *and random computation* *s*_0_ ⇒  ··· ⇒ *s*_*m*_ ⇒  ··· *for 2PartitioN*

$$ P\{s_0\Rightarrow \cdots s_m\Rightarrow \cdots \; \hbox{eventually halts}\}=1. $$

### *Proof*

If cells are active with probability 0.5, then *P*{*σ* is active} = 0.5^||*σ*||^ · 0.5^||*C*−*σ*||^ = 0.5^||*C*||^. In general, if every cell *c* has a probability of activity 1 > *δ*_*c*_ > 0, then there is a *δ* > 0 for which

$$ P\{\sigma \hbox{ is active and } (C-\sigma) \hbox{ is inactive}\} \ge \delta. $$

The probability of random activity taking a computation from *s*_0_ to *t* in at most ||*C*|| steps (as in Theorem 1) is at least *δ* ^||*C*||^. So for *N* = *k*||*C*||, 

$$ P\{s_0\Rightarrow \cdots \Rightarrow s_N \hbox{ has not yet halted}\} \le (1-\delta^{||C||})^k. $$

Since $\lim_{k\rightarrow\infty}(1-\delta)^k = 0,$ the probability of never halting is 0.□

Of course this does not mean that every computation on a bipartite network halts. There are infinitely many non-halting computations—just start with a non-halting *s*_0_ and an edge *ab* ∈ *E* with *s*(*a*) = *s*(*b*), then exclude *a*, *b* from $\sigma_0,\ldots\sigma_m,\ldots$. Something similar is seen in basic measure theory—μ([0,1]) = 1 and μ(*R*) = 0 for the set *R* of rationals, so

$$ P\;\{\hbox{a random}\; 0 \le x \le 1\;\hbox{is irrational}\}=\mu([0,1])-\mu(R)=1.0 $$

and still there are infinitely many numbers that are not irrational. Calculus, with infinite decimals as a metaphor for infinite computations and the set measures as metaphor for probability, is a part of my conceptual framework for amorphous computations.

In the space of all infinite computations, only three things are needed to deduce that some property (say halting) occurs with probability 1. First, every finite computation must have extensions with that property. Second, there must be an upper bound on the number of extra steps to get these extensions (this is a consequence of the finiteness of *C* and *Q*). Third, once an extension with the desired property is reached, every extension of the successful extension will have the property (true for halting states).

### **0,1-Lemma**

*Let* *A* *be a process and* $\Upomega$ *be a property of computations. If (1) extensions of finite computation with property* $\Upomega$ *also have* $\Upomega$, *and (2) every finite computation has an extension with property* $\Upomega$; *then with probability 1*, *A*′*s* *computations eventually have property* $\Upomega.$

There may be infinitely many computations which never satisfy $\Upomega$, but this lemma tells us that we will never see them. The *fairness condition* stated in Section 2 of Aspnes and Ruppert (2007) describes the expected non-determinism in a way similar to this Lemma, then later states *“an execution will be fair with probability 1”*. This paper, which came to my attention from my referees, seems to be moving toward the same measure-theoretic view of non-determinism used here.

## BeatniKs and 3PartitioN

Here we have two simple variations on 2PartitioN. The first, BeatniKs, is a relaxed version which can have halting states which are not accessible, and so this process has a probability of halting which is strictly intermediate between 0 and 1. The second, 3PartitioN solves the NP-complete problem of identifying 3-partite graphs.

**Beatniks** After World War II people who tried to be different might be called “beatniks”. Could everyone be a beatnik? Let *Q* = {0, 1}, *Q* ^+^ = *Q*, 

$$ \alpha(value,input) = 1-input $$

and for multisets *M* = *s*[*E*(*c*)]

$$ input(M) = \left\{\begin{array}{ll} 0 & \hbox{if most of the values in } M \hbox{ are } 0, \\ 1 & \hbox{ if most of the values in } M \hbox{ are } 1, \\ random(\{0,1\}) & \hbox{otherwise,} \end{array}\right. $$

where the random choice is evaluated every time that *c* is active. This process has halting states on networks which are bipartite or almost bipartite. Let *Kn* denote the complete graph on *n* vertices. On *K*(2*m*) a state *s* is halting if and only if ||*s* ^−1^(0)|| = *m*, so *K*6 has 20 halting states. $K3,K5,\ldots K(2m+1)$ have no halting states. For *i* > 2, *Ki* is not bipartite.

### **Theorem 4**

**BeatniKs May Not Halt** *There are no halting states on complete networks with* 2*m* + 1 *cells. Every tree with more than one cell has halting states. However, even when halting states exist they may be* ⇒-*inaccessible from other non-halting states. When such halting states exist the probability of halting is strictly between* 0 *and* 1.

### *Proof*

Every state *s* on an odd complete network *K*(2*m* + 1), will have at least *m* + 1 cells of a given value—say 0. For any 0-valued cell *c*, *s*[*E*(*c*)] has at least half of its values equal to *s*(*c*) and so *input* = 1 − *s*(*c*) or *input* = *random*({0, 1}), so *c* is unstable. At least half of the cells in any state on *K*(2*m* + 1) are unstable. There are no halting states.

Given a tree, construct a halting state by assigning 0 to an arbitrary first cell, then 1 to its neighbors, then 0 to their neighbors, etc.

A binary tree *T*7 = [*w*, [*u*_1_, [*v*_1_, *v*_2_ ] ] , [*v*_3_, [*u*_2_, *u*_3_]]] on *C* = {*u*_1_,…*v*_1_,…,*w*} has four never-halting states *r*_1_, *r*_2_, *r*_3_, *r*_4_ defined by

$$ r_1=[0,[0,[1,1]],[1,[0,0]]],\quad r_2=[1,[0,[1,1]],[1,[0,0]]], $$

*r*_3_ = 1 − *r*_1_ and *r*_4_ = 1 − *r*_2_. In each state, *w* is unstable, while the *u*_*i*_ and *v*_*j*_ are stable. Sets {*r*_0_, *r*_1_} and {*r*_3_, *r*_4_} are closed under ⇒. Still, being a tree, *T*7 has halting states *t*_1_ and *t*_2_ = 1 − *t*_1_. These halting states are inaccessible from *r*_0_, *r*_1_, *r*_2_, *r*_3_. Initial states could be halting or never-halting and so, on such trees,

$$ 0<P\{s_0=t_1\;\hbox{or}\;s_0=t_2\} \le P\{\hbox{Beatniks halts on}\;T7\}\ldots \le 1-P\{s_0=r_1 \;\hbox{or} \ldots \;\hbox{or}\; s_0=r_4\}<1. $$

□

It is not uncommon to see *“something happens with probability 1”* or, *“… with probability 0”*. But in Beatniks on trees like *T*7 the probability of halting is strictly between 0 and 1. This suggests that our work could go beyond traditional 0,1-laws and into analysis 6 using Lebesgue integration 7 of real functions on the product space $(Q^C)^\mathbb{N}$ with measure μ (Sect. 2).

**3PartitioN** Using a transition function *α* on *Q* = {0, 1, 2} which allows a cell *c* to be stable if $s(c)\notin s[E(c)],$ tripartite graphs can be partitioned in a way similar to 2PartitioN on bipartite graphs. Although they are not what we think of as being amorphous, every circuit $C2, C3,\ldots Cm,\ldots$ has a halting state. 8 While complete graphs $K4, K5,\ldots Kn,\ldots$ have no halting states. 9 Tripartite networks have at least six halting states.

This process is interesting because the problem of finding a partition for a tripartite graph is NP-complete (Garey and Johnson 1979). And so our eventual proof that 3PartitioN halts on tripartite (*C*, *E*) could be taken as a demonstration of the computational power of this model. Each network represents one instance of this family of problems. Since networks are free variables, one may think of them as being the input data structures. There is little intelligence in 3PartitioN. Instead this process is executing a blind search of the state space (which will take exponential time) until a state satisfying a simple test is reached. However, 2PartitioN’s global action is *not* NP-complete.

Seven types of neighborhoods are described *Q* ^+^ = {0, 1, 2, 01, 02, 12, 012}. Let

$$ \alpha(value,input) = \left\{\begin{array}{ll} (value+1)\ mod\;3 & \hbox{if} value \hbox{is in} input \\ value & \hbox{otherwise,} \end{array}\right. $$

$$ input_s(c) = \left\{\begin{array}{ll} 0 & \hbox{if only}\;0\;\hbox{is present in}\;s[E(c)], \\ 1 & \hbox{if only}\;1\;\hbox{is present in }s[E(c)], \\ 2 & \hbox{if only}\;2\;\hbox{is present in}\;s[E(c)], \\ 01 & \hbox{if}\;0,1\;\hbox{are present in}\;s[E(c)],\;\hbox{but not}\;2, \\ 02 & \hbox{if}\;0,2\;\hbox{are present in}\;s[E(c)],\;\hbox{but not}\;1, \\ 12 & \hbox{if}\;1,2\;\hbox{are present in}\;s[E(c)],\;\hbox{but not}\;0, \\ 012 & \hbox{otherwise.} \end{array}\right. $$

### **Theorem 5**

**Halting for 3PartitioN** *If* (*C*, *E*) *is tripartite, then for every* *s*_0_ *there exists a halting computation* $s_0\Rightarrow_{\sigma_0} \cdots \Rightarrow_{\sigma_{N-1}}s_N\ldots$ *with* *s*_*N*_ *appropriately partitioning* (*C*, *E*) *and* *N* ≤ 2||*C*||. *3PartitioN almost always (i.e., with probability* 1) *halts on tripartite* (*C*, *E*).

### *Proof*

Let (*C*, *E*) have a halting state *t*. If *s*_*m*_ is not halting, let

$$ \sigma_m=\{c\mid s_m(c)\ne t(c)\hbox{ and c is unstable in } s_m\} $$

and $s_m \Rightarrow_{\sigma_m}\;s_{m+1}$. Since unstable cells appear as edge pairs, *σ*_*m*_ is non-void. No cell *c* can change its value more than twice before it matches *t*(*c*) and is dropped from *σ*_*m*_. So, the process halts (possibly at *t*) in at most $2\|C\|$ steps. □

At first glance, this theorem seems to say that this NP-complete problem can be solved in $<2\|C\|$ steps. Obviously false. Remember that we started with an answer then designed a computation to lead us to an answer. We didn’t have to use a halting state in the proof of halting for 2PartitioN. So it is no surprise that this computation is short and to the point. The hard part is to find *t*. If we could always guess the right computation then tripartition would be easy, and we would have something like *P* = *NP*. 10

Non-determinism plays a key role in this proof. 11 There are many ways to see that randomness can add real power to algorithms. Another is based on comparing the asynchronous state-transition graph (*Q* ^*C*^, ⇒) to its synchronous subgraph (*Q* ^*C*^, ⇒_*C*_) which is often not even connected. Since computations correspond to paths through their corresponding graph the possibilities for asynchronous computations are far greater than for synchronous. Recently, Nazim Fates has made a similar point *“Randomness Helps Computating”* for cellular automata [11].

## The state transition graph (*Q*^*C*^, ⇒, *T*)

Computations can be viewed as paths through the state space. Asynchronous activity often gives computations access to nearly every state.

From an automaton (*Q*, *Q* ^+^, *α*, *input*) for cells and a network (*C*, *E*) defining neighborhoods, (*Q* ^*C*^, ⇒) is the *state transition graph*. The state-transition graph may contain halting states which eventually stop random paths. For other processes, computations may be trapped by *attractors*—minimal disjoint non-void subsets of *Q* ^*C*^ which are closed under ⇒. Attractors are seen in HearT, HearT* and SpotS3.

2PartitioN is an unrestricted search which eventually stumbles into a halting state. Such searches take time which is exponential in $\|C\|:$ add one cell to *C* and the number of states (and the expected search time) increases by a factor of ||*Q*||. Search time is on the order of ||*Q*||^||*C*||^. A computation could reach a state *s* one cell-value away from a halting state and still wander away from the solution losing all of the ground gained. This is the nature of the undirected random search.

$U(s)=||\{c\ |\ c\; \hbox{is unstable in }\; s\}||$ determines a gradient on the state space leading down to halting states. Giving a process the ability to move down such a gradient will dramatically improve the expected halting time; ideally, while preserving the power of asynchronous activity. This must be done locally for the process to remain amorphous.

The state space also provides a framework for algebraic calculations. From activity probabilities *p*(*σ*), we can define transition probabilities

$$ P\{s\Rightarrow t\} = P_s(s, t) = \sum_{s \Rightarrow_{\sigma}t}p(\sigma). $$

Expected values can be defined and calculated. For example, the expected halting time of computations starting at *s*, *h*(*s*) (here defined on states *s*, not computations ${\overline{s}}$ as in Sect. 4) is defined recursively by equations

$$ h(s) = 1+\sum_t P\{s\Rightarrow t\} h(t) \ \hbox{ and } \ h(r) = 0, $$

for unstable *s* and halting *r*. If halting states exist these equations may be solved. Using the uniform probability $q(s)=\|Q\|^{-\|C\|}$ that *s* is initial, the expected halting time for the process is

$$ E[ steps\; to\; halting\; from\; s_0] = \sum_s q(s) h(s). $$

This can be expressed by an equation using the transition matrix *T*.

Imagine states (linearly ordered, or with integer codes) as indices for vectors and matrices. Define the transition matrix *T*, by

$$ T_{t,s} = P\{s\Rightarrow t\}. $$

Probability vectors ${\overline q}$ are column vectors indexed over *Q* ^*C*^. Let ${\overline q}_s=q(s)$ be the probability that *s* is an initial state. Then,

$$ {\overline {qm}} = T^m{\overline q} \hbox{ is the distribution } {\overline {qm}}_t = P\{t=s_m\} $$

that *t* is the *m*th state of a random computation. Examples of these algebraic calculations are given in Sect. 6.

Every process involves three graphs—the cell-communications graph (*C*, *E*), the value-transition graph (*Q*, →) defined by *α*, and the state-transition graph (*Q* ^*C*^, ⇒). A state *s*:(*C*, *E*)→ (*Q*, →) is a *homomorphism* if it preserves edges—i.e., if

$$ cd\in E\; \hbox{implies}\; s(c)\rightarrow s(d)\; \hbox{or}\; s(d)\rightarrow s(c). $$

In 2PartitioN, every state is a homomorphism because (*Q*, →) is complete.

Homomorphisms are similar to continuous functions. Homomorphisms also exist between (*C*, *E*) and itself, or (*Q* ^*C*^, ⇒) and itself. In these cases, a one-one homomorphism is an *automorphism*. $\mathcal{H}\subset Q^C$ is the set of states which are homomorphisms. Any minimal non-void subset of *Q* ^*C*^ which is closed under ⇒ is an *attractor*. Often, attractors are subsets of $\mathcal{H}$—e.g., HearT and 2PartitioN. On *C*4, HearT has one attractor which contains 168 homomorphisms (mechanically counted).

Now, in addition to infinite branches in a ⇒-tree (Sect. 2), and decimals in [0, 1], we have paths through the state graph (*Q* ^*C*^, ⇒, *T*) as metaphors for computation.

## Four programming styles

2PartitioN may be thought of as a random search mechanism with a definition (in local terms) of a halting state. Search continues in response to a failure of a state to satisfy the definition. 2PartitioN* is a gradient-descending process whose computations tend to move down the instability gradient of (*Q* ^*C*^, ⇒, *T*) until the definition is satisfied. A third style can be seen in **2PartitioN** ^#^—a non-homogeneous. 12 version of 2PartitioN which never activates a given cell. Search is now restricted to states extending the given cell’s original value. This is motivated by the idea of a crystal growing from a “seed”. A fourth style uses control on activity. In 2PartitioN^*w*^, cell activity occurs in a wave rolling through the network. A wave of activity is used by Turing (1952) in his solution to the leopards’ spots problem.

2PartitioN* For the instability gradient, we might try

$$ input(M) = \left\{\begin{array}{ll} 0, & \hbox{if most values in }M\hbox{ equal 0}, \\ 1, & \hbox{if most values in }M\hbox{ equal 1}, \\ random(\{0,1\}), \ & \hbox{otherwise;} \end{array}\right. $$

and *α*(*state*,*input*) = 1 − *input*. But this process fails on the tree T7 of Theorem 4. The correct definition follows.

Let *P*{*M* = 0} be the fraction of *M*’s values equal to 0, define

$$ input(M) = \left\{\begin{array}{ll} 0, & \hbox{ with probability }P\{M=0\}, \\ 1, & \hbox{ with probability }1-P\{M=0\}, \end{array}\right. $$

and *α*(*state*, *input*) = *input*. For *s*_0_ on T7, {*s*_0_,*s*_1_} is no longer closed under ⇒. A state *s* is halting if and only if

$$ P\left\{s[E(c)]=s(c)\right\} = 0 \quad \hbox{ for each }c, $$

which is equivalent halting for 2PartitioN. But is 2PartitioN* faster?

Using the algebraic methods described in Sect. 5 on C6 with activity probabilities *p*(*c*) = 2^−1^ and *Q* ^*C*^ indexed as follows  we calculate 13 transition probabilities for 2PartitioN* (2PartitioN) as…

$$ \begin{array}{ll} P^*\{s1\Rightarrow sk\} =\frac{16}{2^{10}} & (=\frac{16}{2^{10}}), \\ P^*\{s2\Rightarrow s4\} =\frac{24}{2^{10}} & (=\frac{32}{2^{10}}),\\ P^*\{s2\Rightarrow s6\} =\frac{72}{2^{10}} & (=\frac{32}{2^{10}}), \\ P^*\{s2\Rightarrow s22\}=\frac{72}{2^{10}} & (=\frac{32}{2^{10}}),\\ P^*\{s4\Rightarrow s1\} =P^*\{s4\Rightarrow s22\}=\frac{9}{2^{10}} & (=\frac{16}{2^{10}}), \\ P^*\{s4\Rightarrow s2\}=\frac{27}{2^{10}} & (=\frac{16}{2^{10}}), \\ P^*\{s22\Rightarrow s22\}=1 & (=1). \end{array} $$

The expected halting times *h*(*s*) for 2PartitioN* are defined by equations

$$ \begin{array}{l} h(s1)=1+(\frac{1}{2^6}h(s1)+\cdots+\frac{3^4}{2^{10}}h(s4)+\cdots), \ldots \\ h(s4)=1+(\frac{3^4}{2^{10}}h(s4)+\cdots+\frac{3^3}{2^{10}}h(s7)+\cdots +\frac{3^2}{2^{10}}h(s21)+\cdots),\ \ldots \\ h(s22)=0, \ldots etc. \end{array} $$

Equivalently, working in matrices and vectors with these indices, T*’s values include

$$ T^*_{2,1}=T^*_{4,1}=T^*_{22,1}=\frac{16}{2^{10}}, \quad T^*_{1,4}=T^*_{22,4}={9\over {2^{10}}}, \quad T^*_{22,22}=1, \quad T^*_{n,22}=0 \ (n \ne 22),\ldots$$

with the symmetry $T_{j,i}^{\ast} = T_{65-j,65-i}^{\ast}$. The expected halting times, for *t* halting and *s* non-halting, are defined using matrices as  where  is the vector of all 1’s and ${\overline h}$ is the vector of expected halting times—e.g., ${\overline h}_{22}=0$—all indexed over *Q* ^*C*^. Solve these equations, then for the initial state use ${\overline q}=<\cdots 2^{-6},\cdots>$ to get

$$ \begin{aligned} \hbox{E(2PartitioN halting time)} &=16.57, \\ \hbox{E(2PartitioN* halting time)} & = 9.39, \\ \hbox{E(2PartitioN}^{\#}\; \hbox{halting time)} & = 14.95. \end{aligned} $$

Add the edge 14 to *C*6 to get a network with slightly higher average cell degree—call it C4C4. Repeat the algebra and the speed-ups are better

$$ \begin{aligned} \hbox{E(2PartitioN halting time)} &= 22.27, \\ \hbox{E(2PartitioN* halting time)} &= 8.21, \\ \hbox{E(2PartitioN}^{\#}\; \hbox{halting time)} &= 12.97. \end{aligned} $$

So algebraic analysis shows a significant speedup, on these small networks. I have no algebra for 2PartitioN^w^, but simulations suggest

$$ \hbox{E(2PartitioN}^w \hbox{halting time)}\approx \hbox{E(2PartitioN}^{\#}\;\hbox{halting time)} $$

and this method of waves has been demonstrated by Coore (1999).

### **Theorem 6**

2PartitioN* Halting On a bipartite network, 2PartitioN* almost always halts.

### *Proof*

Similar to the proof of halting for 2PartitioN except that (due to the non-determinism) ∂*X*_*n*_ and ∂*X*_*n*+1_ may not be disjoint, so $X_0\subseteq \cdots X_m\subseteq X_{m+1}\subseteq \cdots C.$

If *k* is the maximal degree of cells in (*C*, *E*), then the expected number of times that an *s*-unstable cell *c*, must be active before *s*(*c*) changes is at most $k=\frac{1}{k}1+\frac{1}{k}\frac{k-1}{k}2+\cdots+\frac{1}{k}(\frac{k-1}{k})^j(j+1)+\cdots.$ For some $\epsilon > 0,$ these computations will halt in *k*||*C*|| steps with probability $\ge \;\epsilon.$ And so, computations of length *mk*||*C*|| fail to halt with probability $< (1- \epsilon)^{m}.$ Over the long run, these processes fail to halt with probability $0=\lim_{m=\infty}(1-\epsilon)^m.$□

### **Theorem 7**

**2PartitioN** ^#^ 2*PartitioN* ^#^ *halts on bipartite nets*.

### *Proof*

The original proof for 2PartitioN works here.□

For 3PartitioN*, I have no gradient. A successful gradient for 3PartitioN would enable the process to halt in (expected) polynomial time—polynomial in ||*C*|| · log_2_(||*Q*||).

## Patterns in attractors

Three versions of SpotS, for spatial patterns, and HearT*, for temporal patterns, are developed in this section. SpotS*i* were inspired by Turing (1952).

**SpotS1** has values *r* for red and *y* for yellow. A red cell is not stable until its neighbors are all yellow. A yellow cell is not stable until it has a red neighbor. If reds are not tightly packed, then yellow surrounds a yellow cell, making it unstable, it becomes red.

### **Theorem 8**

**SpotS1 Halts** *On every* (*C*, *E*), *SpotS1 will halt*.

### *Proof*

First, build a halting state from a first red cell *c*_1_ by coloring ∂{*c*_1_} yellow, then choose a second red cell from *c*_2_ ∈ ∂({*c*_1_} ∪ ∂{*c*_1_}) and on until a halting *t* has been constructed. Second, use *t* to construct a computation satisfying the necessary three properties (Sect. 3), finally conclude that almost every computation halts. □

**SpotS2** This process wraps each red cell in a ring of blue cells and then constructs a minimal yellow background, with some white where unavoidable. White surrounded by white is unstable and becomes red. We require (1) reds to have blue neighbors only, (2) blues to have exactly one red neighbor, (3) yellows to have at least one blue neighbor, and (4) whites to have yellow neighbors but no blue neighbors. The first requirement guarantees that yellows have no red neighbors. Given *Q* = {*r*, *b*, *y*, *w*} and *Q* ^+^ = *Q* ∪ {*o*}, define

$$ \alpha(value,input)=\left\{\begin{array}{ll}input,\ &\hbox{if}\; input=r,b,y,w, \\ random(\{r,b,y\}),\ &\hbox{ otherwise,} \end{array}\right. $$

$$ input(M)=\left\{\begin{array}{ll} r,\ &\hbox{if}\; M \hbox{ contains only } b, \\ b, &\hbox{if}\; M \hbox{ contains exactly one } r, \\ y,\ &\hbox{if}\; M \hbox{ contains at least one } b, \\ w, &\hbox{if}\; M \hbox{ contains only } y, \\ o,\ &\hbox{ otherwise.} \end{array}\right. $$

**Fig. 2:**
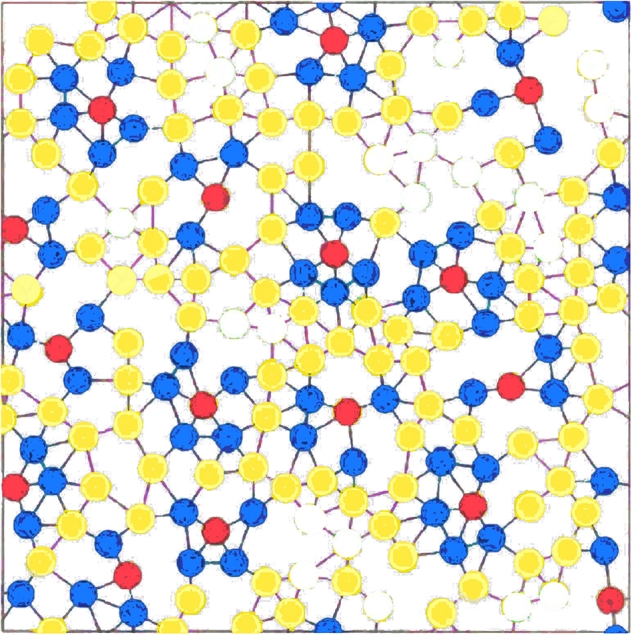
A state from an attractor for SpotS3

Halting states exist on every network, so computations eventually halt. The only flaw is that blue rings, for different red cells, are allowed to touch. SpotS3 corrects this problem (Fig. 2).

**SpotS3** detects touching blue rings. Dynamic indices (hidden variable) are introduced *Q* = {*r*1, *r*2, *r*3, *b*1, *b*2, *b*3, *y*, *w*}. Invisible changes $r1\rightarrow r2\rightarrow r3\rightarrow r1\rightarrow\ldots$ and $b1\rightarrow b2\rightarrow b3\rightarrow b1\rightarrow\ldots$ advance red indices while neighboring blues keep up. So that a stable blue *bi* will be at most one behind its *rj* center—i.e., *j* = *i* or *j* = (*i* + 1)mod3. Touching blue rings follow different red centers and eventually have an index conflict—i.e., a *bi* will see its *rj* neighbor and a *bk* neighbor *k* = (*j* + 1)mod3. This is indicated by *input* = *o*. A destabilized spot is deconstructed.

Let *Q* ^+^ = *Q* ∪ {*o*} then, for *j* = (*i* + 1)*mod* 3 and *k* = (*j* + 1)*mod* 3,

$$ input(M)=\left\{\begin{array}{ll} rj &\hbox{if}\; M\; \hbox{contains}\; bi \hbox{ and possibly } bj \hbox{ but nothing else, } \\ bj &\hbox{if}\; M \hbox{ contains only } rj,bi,bj,y, \\ y &\hbox{if}\; M\; \hbox{contains blues and whites but no reds, } \\ w & \hbox{if}\; M\; \hbox{contains only}\;y, \\ o  &\hbox{otherwise,} \end{array}\right. $$

$$ \alpha(value,input)=\left\{\begin{array}{ll} random(\{r1,r2,r3,b1,b2,b3,y\}), & \hbox{if}\; input=o, \\ input, &\hbox{otherwise.} \end{array}\right. $$

**Fig. 3:**
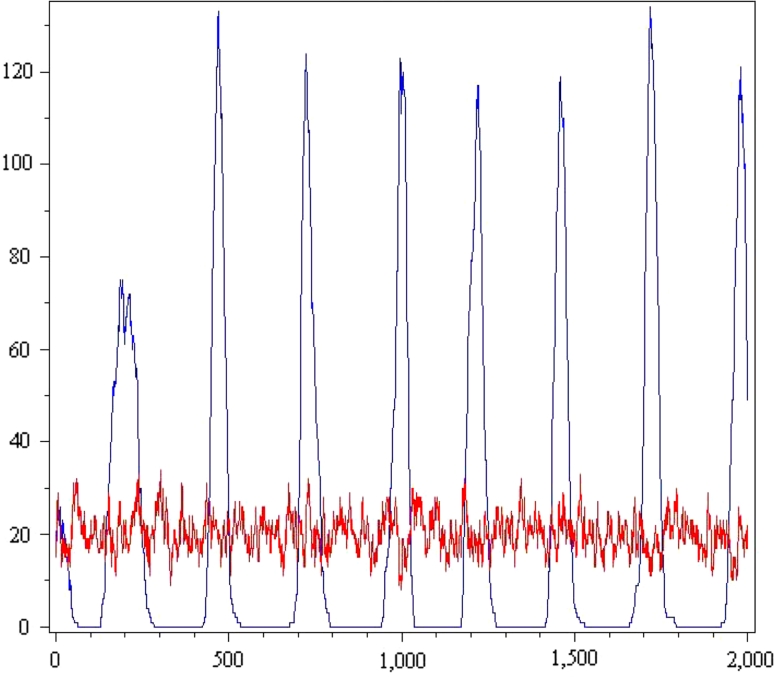
Gradient-directed wave formation in HearT*, but not HearT

This process eventually settles into an attractor whose states have fixed color assignments and changing indices on red and blue values (Fig. 3).

HearT* is a gradient following process which develops a *temporal* pattern by coordinating cellular oscillators. HearT* is not intended to halt, instead computations enter attractors composed of states which are homomorphisms of the network into (*Q*, →). The gradient approaching these attractors is determined by the number of edges broken by *s*.

Given *Q* = {0, 1, …*c*, 9} and *Q* ^+^ = *Q*, define

$$ \alpha(value,input) = (value+input)\;\hbox{mod}\;10, $$

$$ input_s(c) =   \left\{\begin{array}{ll}    0 & \hbox{if}\; B_s(c) < B_{s^c}(c), \\    1 & \hbox{otherwise. } \end{array}\right. $$

$B_s(c) = \|\{d\in E(c)\ |\;\hbox{neither}\;s(c)\rightarrow s(d),\;\hbox{nor}\,s(d)\rightarrow s(c)\}\|$ counts edges at *c* broken by *s*. When applied to

$$ s^c(d) =   \left\{\begin{array}{ll}    s(d)      & \hbox{if }d \ne  c, \\    (s(c)+1)\;\hbox{mod}\;10 & \hbox{otherwise.}   \end{array}\right. $$

$B_s^{c} (c)$ counts edges broken by advancing *s*(*c*). Once HearT* reaches a homomorphism, subsequent states will be homomorphisms. The original HearT differs in that

$$ input_s(c) =   \left\{\begin{array}{ll}    0 & \hbox{if}\; B_s(c)=0\; \hbox{and}\; 0 < B_{s^c}(c), \\ 1 & \hbox{otherwise.}\end{array}\right. $$

This removes the B-gradient used to guide HearT* to an attractor. HearT and HearT* have the same attractors But before entering an attractor *B*_*s*_ occasionally increases—even for HearT*. 14

In the plot at Fig. 3, we see computations for HearT* and HearT on the 200-cell network shown in Fig. 1. Both processes begin at the same state, but HearT*’s search (blue) reaches an attractor and begins coordinated oscillations in fewer than 500 steps, while HearT is still searching (red) after 2,000 steps. This plot shows ||*s*_*n*_^−1^(0)|| as a function of *n*.

**Fig. 4:**
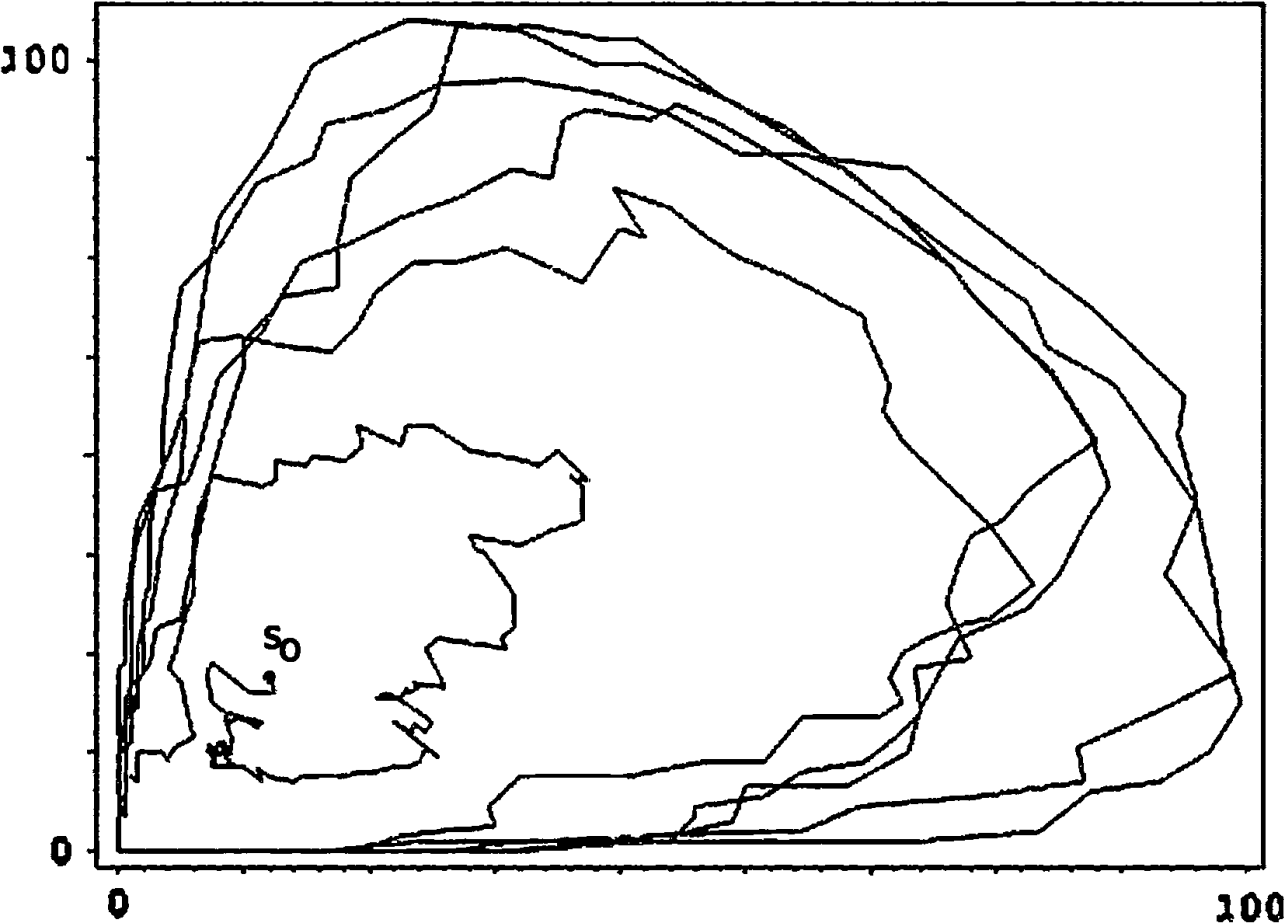
$$\hbox{HearT}^\ast: $$ an (*x*_*m*_, *y*_*m*_) orbit *x*_*m*_ = ||*s*_*m*_^−1^(9)||,*y*_*m*_ = ||*s*_*m*_^−1^(0)||

The table below gives another view of self-organization by HearT*. The initial state *s*_0_ has about 20 cells for each value. At step 200, cells are competing for values of 1 and 4. After step 400, the colony settles in on a single value.  Finally, morphogenesis for HearT* is seen in Fig. 4 as an orbit through a space of value-count pairs (*x*_*m*_, *y*_*m*_) = (||*s*_*m*_^−1^(9)||, ||*s*_*m*_^−1^(0)||) for *m* = 0…1,500. A counter-clockwise, outward spiral begins at *s*0 near (20,20). At the bottom, motion to the right, is due to increasing the number of cells whose value is 9. Then, motion to the top-left shows cells moving from 9 to 0. Increased organization is seen as increased radius for the spiral.

Strogatz (2003) has written on systems of oscillators which vary continuously and so are not as simple as our finite-state automata. He mentions a conjecture by Peskin asserting that “[global] synchronization will occur even if the oscillators are not quite identical”. Given this, we might ask “if cells in HearT are reduced from ten values to nine values, or increased to eleven; will the process continue to oscillate?”. I conjecture “yes”, as long as the nine’s don’t touch the ten’s.

HearT* is robust in the face of colony growth, and cell death. Living samples of cardiac tissue develop a heart-beat without centralized control. 15 An interesting experiment, after such a colony has self-organized and begun to beat, would be to cool (thus slow) the cells on one half of the culture but not the other. If cells in the warmer half slow their beat to match their cooler mates, then these living tissues are, like HearT, behaving to preserve continuity of values with neighbors—just like our homomorphisms (Fig. 4).

## Algebraic calculations

**OddParitY** is defined on *Q* = {0,1} with *Q* ^+^ = *Q* by

$$ \alpha(value,input)=1-input $$

$$ input_s(c) =   \left\{\begin{array}{ll}    0 & s[E(c)]\;\hbox{contains an even number of }1s, \\    1 & \hbox{otherwise.}   \end{array}\right. $$

An *s* is halting if and only if every *s*[*E* ^+^(*c*)] has odd parity, or

$$ \left(\sum\limits_{d\in E^+(c)} s(d)\right)\;\hbox{mod}2 = 1. $$

On C6, OddParitY has four halting states: 111111, 100100, 010010, 001001 ; C10 has one, and K4 has eight. But, based on previous proofs, it is hard to imagine a general proof of halting for this process.

### **Theorem 9**

**OddParitY Halting** *Halting states exist on every* (*C*, *E*).

### *Proof*

(This proof is based on Sutner (1989).) Work in linear algebra with *Q* ^*C*^ as the vector space, mod2 arithmetic in *Q* and dimensions indexed by $c\in C.$ Halting is defined by  where *E* is the network’s adjacency matrix, 16 *I* is the identity matrix and  is the all-1s vector.

If (*E* + *I*) is invertible then  otherwise *range*(*E* + *I*)≠ *Q* ^*C*^ and so

$$ kernel(E+I)=range(E+I)^\perp \ne \emptyset. $$

If  then  has a solution *s* which is halting. Now prove that  is in the range.

Let  be the all-0s vector and  be the subvector of *v* formed by restricting indices to *C*_*t*_. For  in *kernel*(*E* + *I*) let  (*C*_*t*_, *E*_*t*_) is formed by deleting *t*’s 0-valued cells, so  (from *t* being in the kernel) implies  then, since  every *d* has odd degree.

Edges are counted twice when summing cell-degrees, so with ||*E*_*t*_|| equal to the number of edges in *E*_*t*_

$$ \sum_{d\in C_t} degree_{E_t}(d)=2\left\|E_t\right\|=0, $$

since *degree*_*E*_*t*_(*d*) is always odd, $\left\|C_t\right\|$ is even,  and  Finally, OddParitY always has a halting state.□

**Fig. 5:**
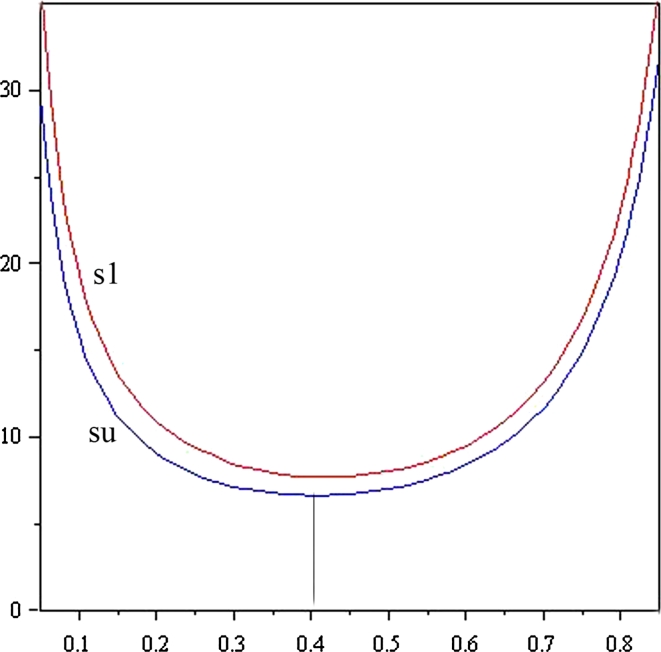
Expected halting times, *E*(*p*), as a function of activity *p*

Sutner’s proof has (*C*, *E*) as a free variable and so it is not sensitive to the size of the network. I find this proof’s power surprising (Fig. 5).

In 2009 and 2010, my students Arash Ardistani and Robert Lenich independently calculated the expected halting time as a function *E*(*p*) of *p* (the uniform probability of cell activity, previously denoted *δ*) for processes on C4 and several other small networks. Here is how it was done with C4-states $s1,\ldots s16$ indexed as in Sect. 6 The expected halting time *hi* from a non-halting *si* satisfies

$$ hi = 1 + \sum_{j=1\ldots 16}P_c\{si\Rightarrow sj\}\cdot hj\ $$

and *hj* = 0 for halting *sj*, where for example *P*_*c*_{*s*3 ⇒  *s*4} = *p* ^2^(1 − *p*)^2^ + *p*(1 − *p*)^3^ = *p*(1 − *p*)^2^, etc. Solve these 16 equations for the *hi* to get equations such as

$$ \ldots,\ h3=\frac{-6p^3+15p^2-14p+6}{2p(2p^2-3p+2)(1-2p+p^2)},\ \ldots. $$

Now, use these *p*-terms in $$E(p)=\sum_{i=1}^{16}\frac{1}{16} hi \ $$ to get

$$ \begin{aligned} E(p) & = {3\over {32}}\frac{-7-20*p^2+8*p^3+18*p}{(2*p^4-7*p^3+10*p^2-7*p+2)*p} -{3\over {32}}\frac{6*p^3-15*p^2+14*p-6}{p*(-1+p)^2*(2*p^2-3*p+2)} \\ & \quad- {5\over {32}}\frac{6*p^3-15*p^2+14*p-6}{p*(2*p^2-3*p+2)*(1-2*p+p^2)} -{3\over {32}}\frac{-7-20*p^2+8*p^3+18*p}{p*(2*p^2-3*p+2)*(1-2*p+p^2}. \end{aligned} $$

Which is optimized by solving $0=\frac{d}{dp} E(p)$ for *p*. The solution *p* = 0.4064 gives *E*(0.4064) = 6.636. *E*(*p*) is plotted above for 2PartitioN computations on C4 starting (1) from *s*_0_ = [0, 0, 0, 0], and (2) from a (uniformly) random initial state *s*_0_. We see

$$ \lim_{p\rightarrow 0}E(p) = \infty \quad \hbox{and}\quad \lim_{p\rightarrow 1}E(p) = \infty $$

because *p* = 1 corresponds to synchronous activity and *p* = 0 corresponds to no activity, neither of which solves the problem. So the fastest process is asynchronous (i.e., 0 < *p* < 1). All other processes on all other nets showed the expected halting time going to infinity as *p* approached 0, and (most of processes) as it approached 1.

## Limits on amorphous computability

Imagine a process, call it **ElectioN**, which eventually halts with exactly one cell in a computed value. I will prove that no program exists which computes the desired state in the absolutely amorphous model. This non-computability result is analogous to the non-computability, by Turing Machine, of the halting problem’s solution. The problem of election in a distributed process has been discussed since Le Lann (1977) and recently in dynamical amorphous processes 17 by Dereniowski and Pelc (2012).

**Fig. 6:**
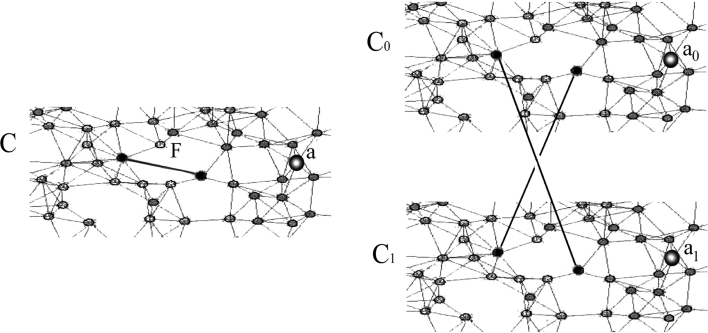
A stability-preserving construction

In the following, a halting state *s* for ElectioN on (*C*, *E*) is assumed to exist. Then a larger network (*C*′, *E*′) is constructed with a halting state *s*′ for which $\|s^{-1}(a)\|\ne 1$ for every $a\in Q.$ By contradiction, no program exists for computing ElectioN’s halting states in an absolutely amorphous environment (Fig. 6).

### **Theorem 10**

**ElectioN is not Absolutely Amorphous** *No program exists which, independent of the underlying net, almost always* 18 *halts in a state s with* ||*s* ^−1^(*a*)|| = 1 *for some* $a\in Q.$

### *Proof*

Assume ElectioN has a program, and that (*C*, *E*) has an *s* which is halting for that program. By assumption, ||*s* ^−1^(*a*)|| = 1 for some *a*. Let *C*′ be the set of 0,1-indexed copies of cells in *C*—i.e.,

$$ C^{\prime}=\{c_i\ |\ c\in C\quad  \hbox{and}\quad i=0,1\}. $$

Choose a non-void subset $F\subset E$ which is not a cut-set 19 of (*C*, *E*), then define edges on *C*′ by

$$ E^{\prime}=\{c_0d_0,\ c_1d_1\ |\ cd\in E-F\} \cup \{c_0d_1,\ c_1d_0\ |\ cd\in F\}. $$

(*C*′, *E*′) is connected. Construct *s*′ by *s*′(*c*_0_) = *s*′(*c*_1_) = *s*(*c*) for every $c\in C.$ From the construction, we have

$$ s^{\prime}[E^{\prime}(c_0)] = s^{\prime}[E^{\prime}(c_1)] = s[E(c)] $$

for every $c\in C,$ so $input_{s^{\prime}}(c0) = input_{s^{\prime}}(c1) = input_{s^{\prime}}(c)$ and

$$ \alpha(s^{\prime}(c_0),input_{s^{\prime}}(c_0)) = \alpha(s^{\prime}(c_1),input_{s^{\prime}}(c_1)) = \alpha(s(c),input_s(c)). $$

Thus, *s*′ is halting on (*C*′, *E*′), ||*s* ^−1^(*a*)|| is even for every *a*. No value occurs exactly once. By contradiction, ElectioN is not absolutely amorphous.^20^□

When a unique value or token is needed, it can be provided in *s*_0_; but then the initial state is not random and the process is not absolutely amorphous.

The trick of Theorem 10 can be used to prove that there is no absolutely amorphous process, Not2PartN (a compliment for 2PartitioN), which halts precisely on networks which are not bipartite. And, to prove that there is no absolutely amorphous process, StricT3PartN, which recognizes networks which are tripartite but not bipartite. Proof sketch: assuming that Strict3PartN exists, a halting state *s* exists on *C*3 = ({*a*, *b*, *c*}, {*ab*, *bc*, *ca*}). Join copies *C*3_1_ and *C*3_2_ by crossing the *bc* edges (producing *b*_1_ *c*_2_ and *b*_2_ *c*_1_ in place od *b*_1_ *c*_1_ and *b*_2_ *c*_2_) to construct *C*6, and define a state *t* on *C*6 defined from *s* by *t*(*a*_*i*_) = *s*(*a*), *t*(*b*_*i*_) = *s*(*b*), *t*(*c*_*i*_) = *s*(*c*). Obviously *a*_1_,*a*_2_ are stable, but what about the *b*’s and *c*’s? For $b_1,\ldots$

$$ t(b_1)=s(b), $$

$$ t[E(b_1)]=t[\{a_1,c_2\}]=\{t(a_1),t(c_2)\}=\{s(a),s(c)\}=s[E(b)], $$

$$ \hbox{and }\ \alpha(t(b_1),t[E(b_1)])=\alpha(s(b),s[E(b)])=s(b)=t(b_1), $$

so *t* is stable at *b*_1_. The remaining cells are the same, and so *t* is a halting state. But *C*6 is bipartite - contradiction.

To go further into recursion theory these processes must be expressed in the integer framework of Kleene’s partial recursive functions. I cannot expect to carry everything into recursion theory because the random choice function is not Turing computable. But the deterministic part, of what has been developed here is computable, and since the structures are finite they can be coded 21 eventually into ${\mathbb{N}. }$

## TokeN, GradienT and RaiN

A token is a unique value which moves through a network, from one cell to a neighbor, without being assigned to more than one cell at a time. Assuming that *s*_0_ has $s_0(a)=T\in Q$ for exactly one cell and *s*_0_(*d*) = *q* for all others, TokeN is a process whose computations move *T* randomly through (*C*, *E*) and without duplication. Further, TokeN is shown to be self-stabilizing in the sense of Dijkstra.

**TokeN** First, the movement of *T* is described informally as follows.

1. Neighbors *b*, *c* of *s*(*a*) = *T* cycle $q\rightarrow r\rightarrow s\rightarrow q$ while $s(a)=T\rightarrow T$ as long as *s*[*E*(*a*)] does not contain exactly one *r* (i.e.,receiver), but $s(a)=T\rightarrow T1$ is allowed when the neighborhood contains exactly one *r*. All others, do $q\rightarrow q.$
2. The neighborhood of *s*(*a*) = *T*1 may have changed at the moment of $T\rightarrow T1,$ so $T1\rightarrow T$ (the advance is retracted) if *s*[*E*(*a*)] does not still contain exactly one *r*. But if *s*[*E*(*a*)] still has exactly one *r* = *s*(*b*), then the token holder advances one more step $T1\rightarrow T2=s(a).$ Neighbors of *s*(*a*) = *T*1 never change their value while *s*(*a*) = *T*1, *T*2, so a new *r* cannot appear among neighbors of *a* after *T*1.
3. For neighbors of *s*(*a*) = *T*2, the token is passed to *b* the singular receiver $r \rightarrow T.$ At $a, T2\rightarrow T2$ as long as $T\notin s[E(a)],$ but $T2\rightarrow q$ after *s*(*b*) = *T*.

Now we go back to (1), except that *T* has passed from *a* to *b*.

This process uses values *Q* = {*q*, *r*, *s*, *T*, *T*1, *T*2}; inputs *T*, *qrs*, *r*!*qs* are used in stage 1 and *T*1, *T*2 for stages 2 and 3. Thus *Q* ^+^ = *Q* ∪ {*qrs*, *r*!*qs*}.

$$ \alpha(value,input) = \left\{\begin{array}{ll} r & \hbox{if}\;value=q\hbox{ and }input=T, \\ & \hbox{if}\;value=r \hbox{ and }input=T1, \\ s & \hbox{if}\;value=r \hbox{ and }input=T,\\ & \hbox{if}\;value=s \hbox{ and }input=T1, \\ q & \hbox{if}\;value=s \hbox{ and }input=T,\\ & \hbox{if}\;value=q \hbox{ and }input=T1, \\ & \hbox{if}\;value=q \hbox{ and }input=q,qrs,r!qs\\ & \hbox{if}\;value=T2 \hbox{ and }input=T, \\ T & \hbox{if}\;value=T \hbox{ and }input=qrs,\\ & \hbox{if}\;value=T1 \hbox{ and }input=qrs, \\ & \hbox{if}\;value=r \hbox{ and }input=T2,\\ T1 & \hbox{if}\;value=T \hbox{ and }input=r!qs, \\ T2 & \hbox{if}\;value=T1 \hbox{ and }input=r!qs, \\ value & \hbox{otherwise.} \end{array}\right. $$

$$ input(M) = \left\{\begin{array}{ll} T & \hbox{if}\; M \hbox{ contains } T, \\ T1 & \hbox{if}\; M \hbox{ contains } T1, \\ T2 & \hbox{if}\; M \hbox{ contains } T2, \\ qrs& \hbox{if}\; M \hbox{ contains any of } q,r,s, \hbox{ but the } r \hbox{ is not unique, }\\ r!qs & \hbox{if}\; M \hbox{ has exactly one}\; r,\;\hbox{and possibly}\; q\hbox{'s and}\; s\hbox{'s}, \\    q   & \hbox{otherwise.}  \end{array}\right. $$

Dijkstra (1974) describes a network stabilization problem. Certain actions must be performed serially by the processors in the network. This requires a network procedure which manages a token (which grants the privilege to act). But the network is prone to failures which create a state with more than one token—an unstable state. If TokeN is used as a token manager on Dijkstra’s network then (given enough time) it will, with probability 1, merge excess tokens until only one remains. To prove this imagine a finite sequence of states $s_0,\ldots, s_m$ is which *s*_*m*_ has two *T*s. A finite extension $\ldots s_m \Rightarrow \cdots \Rightarrow s_{m+n}$ can always be found which moves these tokens to a place where they share a neighbor *d*. Then extend the computation to $\ldots s_m\Rightarrow\cdots\Rightarrow s_{m+n}\Rightarrow\cdots\Rightarrow s_{m+n+o}$ which ends with both tokens being simultaneously passed to *d*—thus merging them. There is only one token in *s*_*m*+*n*+*o*_ and by the 0,1-Lemma, this merger of multiple tokens happens with probability 1.

**GradienT**_*N*_ Let $Q=\{0,1,2,\ldots,N-1\}$ up to some large *N*, *Q* ^+^ = *Q* and *input*(*M*) = *min*(*M*)mod*N* and *alpha*(*value*,*input*) = (*input* + 1)mod*N* for *M* = *s*[*E*(*c*)]. Given networks with exactly one cell *r* (for root) inactive, and states *s* having 0 = *s*(*r*) and 0 < *s*(*c*) for *c* ≠ *r*, this process eventually assigns to *c* a value equal to the length of the shortest path connecting *c* to *r*. After value *N* − 1, the counting starts over at 1. Subsequent processes may appear to pass values down-gradient to *r*, by allowing values to be read from up-gradient cells.

**RipplE** given a state $s\in\{0,1,2\}^C,$ signals the presence of a 2 to the rest of the network (imagine 0s) by propagating out 2s, then reducing 2s to 1s, then 1s to 0s—all moving away from the original *s*_0_(*c*) = 2. Values circulate $0\rightarrow 2\rightarrow 1\rightarrow 0.$ With *Q* ^+^ = {0, 1, 2, 01, 12, 02, 012} define

$$ \alpha(value,input)=\left\{\begin{array}{ll} 2 & \hbox{for}\; value=0\; \hbox{and}\; input=2,02,     \\   2 & \hbox{for}\; value=2  \hbox{ and } input=0,02,     \\   1 & \hbox{for}\; value=2  \hbox{ and } input=1,2,01,12,012, \\   1 & \hbox{for}\; value=1  \hbox{ and } input=2,12,     \\   0 & \hbox{for}\; value=0,1 \hbox{ and } input=0,1,01,012,  \\   0 & \hbox{for}\; value=1  \hbox{ and } input=02,      \\   0 & \hbox{for}\; value=0  \hbox{ and } input=12,  \end{array}\right. $$

$$ input(M)=\left\{\begin{array}{ll}   0  & \hbox{ for } M \hbox{ containing only } 0s,   \\   \ldots & \ldots \\   12 & \hbox{ for } M \hbox{ containing only } 1s, 2s, \\ \ldots & \ldots                     \\   012 & \hbox{ for } M \hbox{ containing }0s, 1s, 2s.  \end{array}\right. $$

The first two lines in *α*’s definition apply to cells just before, and at the leading edge of the ripple. In the remaining lines, the presence of a 1 indicates that the ripple has passed.

In **RaiN**, new 2-values spontaneously appear (as input, not as computed values) in states during this computation and trigger their own ripples (like rain drops on a pond). Unfortunately, if they appear near a 1, they will degenerate (third line above) without a ripple. So a pre-2 value 3 will represent the rain drop. We need only add 3 to *Q* and two lines to *α*

$$ \alpha(value,input)=\left\{\begin{array}{ll}   3   & \hbox{ for } value=3 \hbox{ and } input \ne 0, \\   2   & \hbox{for}\; value=3 \hbox{ and } input=0,   \\   \ldots & \hbox{ same as for RipplE } \ldots  \end{array}\right. $$

Input will be blind to 3, and the new 3 is preserved until its neighbors settle down to 0s. RaiN is used in *X*HalT?.

## Composition and other operations

Operations on processes—product, serialization, and composition—are presented by example.

**TokeN** ^2^ is the product of TokeN (*Q* ^*T*^, *Q* ^*T*+^, *α*, *input*) with itself. This construction uses *Q*_*TT*_ = (*Q*_*T*_)^2^ and *Q*_*TT*_^+^ = (*Q*_*T*_^+^)^2^—pairs of values from TokeN. Projections are (*sq*)_1_ = *s* and (*sq*)_2_ = *q* for $sq\in Q_{TT},$ and $M_1=\{v\ |\ vw\in M\},$ etc. for $M\subset Q_{TT}.$ The program is completed by

$$ input^{TT}(M) = [input^T(M_1),input^T(M_2)] \hbox{ for } M\subset Q_{TT}, $$

$$ \alpha^{TT}(value,input) = [\alpha^T(value_1,input_1),\alpha^T(value_2,input_2)]. $$

(*T* and *TT* are used as subscripts on *Q* to avoid confusion with the set exponent *Q* ^*C*^.)

To have tokens move through (*C*, *E*) independently, activity in the first and second dimension must be independent. For *σ* = [*σ*_1_,*σ*_2_] where $\sigma_1,\sigma_2\subset C,$

$$ s\Rightarrow_{\sigma}^{TT}s^{\prime} \hbox{ is } s_1\Rightarrow_{\sigma_1}^T s_1^{\prime}\quad \hbox{and}\quad s_2\Rightarrow_{\sigma_2}^T s_2^{\prime}. $$

Usually, process products are a framework for the exchange of information (between the processes). But not in TokeN^2^.

**SeriaL** *Y* is the token-activated serial-execution of *Y*. This process could as well be called **if-TokeN-then-** *Y*. Information from the first process (the presence of the token at a cell) is used to determine the cell activity which executes *Y*. Since there is only one token, *Y* experiences serial execution.

For example, let *Y* be 2PartitioN, and **T2P** be SeriaL2*PartitioN*. The languages are *Q*_*T*2*P*_ = *Q*_*T*_ × *Q*_2*P*_ and *Q*_*T*2*P*_^+^ = *Q*_*T*_^+^ × *Q*_2*P*_^+^. Transition and input, for $s:C\rightarrow Q_{T2P}$ and *M* = *s*[*E*(*c*)] on (*C*, *E*), are

$$ \alpha^{T2P}(value,input) = \left\{\begin{array}{lll} \left[\alpha^T(value_1,input_1),\alpha^{2P}(value_2,input_2)\right] & \hbox{if}\; value_1=T, \\ \left[\alpha^T(value_1,input_1),value_2\right] & \hbox{ otherwise, }  \end{array}\right. $$

$$ input^{T2P}(M) = \left[input^T(M_1),input^{2P}(M_2)\right]. $$

If both processes are active at *σ* and a cell $c\in\sigma$ holds the token, then

$$ s \Rightarrow_{\sigma}^{T2P}s^{\prime}\; \hbox{if and only if}\; s_1\Rightarrow_{\sigma}^Ts_1^{\prime}\; \hbox{and}\;s_2\Rightarrow_{c}^{2PN}s^{\prime}_2. $$

*X* **HalT?** is the product of *X* with a process which copies the *X*’s old value from *s*(*c*)_1_ to *s*(*c*)_2_ before *X* assigns a new value to *s*(*c*)_1_, and finally RaiN (which spreads news of some *s*(*c*)_1_ ≠ *s*(*c*)_2_ to other cells by *s*(*c*)_3_ = 3). After *X* has a halting state in *s*_1_, this process will have solid 0s in *s*_3_. For RaiN, let *Q*_*R*_ = {0, 1, 2, 3} and *Q*_*R*_^+^ = {0, 1, 2, 3, 01, 02, 12, 123}.

Set *Q*_*XH*?_ = *Q*_*X*_ × *Q*_*X*_ × *Q*_*R*_ and *Q*_*XH*?_^+^ = *Q*_*X*_^+^ × *Q*_*X*_^+^ × *Q*_*R*_^+^, then

$$\alpha^{XH?} (value, input) =  \left\{\begin{array}{ll} \left[\alpha^X(value_1,input_1),value_1,3\right] & \hbox{if}\; value_1 \ne value_2,\\ \left[\alpha^X(value_1,input_1),value_1,\alpha^R(value_3,input_3)\right] & \hbox{otherwise,}  \end{array}\right. $$

where *α* ^*R*^ is RaiN, and

$$ input^{XH?}(M)=[input^X(M_1),0,input^R(M_3)] $$

for $M\subset Q_{XH?}.$ After *X* halts, RaiN will spread 0s to every *s*(*c*)_3_.

(**Y** ∘ **X**) is the composition of processes *Y* and *X* with shared values *Q*_*X*_ = *Q*_*Y*_. It uses *X*HalT? to initiate *Y*. Set *Q*_*Y* ∘ *X*_ = (*Q*_*XH*?_) × *Q*_*Y*_ and treat these values as having projections—$[value_{11},value_{12}]\in Q_X^2,$ $value_{13}\in Q_R, value_2\in Q_Y$ so *value*_1_ = [*value*_11_, *value*_12_, *value*_13_]—then define

$$ \begin{aligned} \alpha^{Y \circ X}(value,input) =\\ \left\{\begin{array}{lr} \left[\alpha^{XH?}(value_{1},input_{1}),\alpha^Y(value_{11},input_{11})\right] & \hbox{if}\; 0<value_{13},\\ \left[\alpha^{XH?}(value_{1},input_{1}),\alpha^Y(value_2,input_2)\right] & \hbox{ otherwise,}  \end{array}\right. \end{aligned} $$

and for *Q*_*Y* ∘ *X*_^+^ = *Q*_*XH*?_^+^ × *Q*_*Y*_^+^ and $M\subset Q_{Y\circ X},$

$$ input^{Y\circ X}(M) =  \left\{\begin{array}{ll} \left[input^{XH?}(M_1),input^Y(M_{11})\right] & \hbox{if } 0<value_{13}, \\   \left[input^{XH?}(M_1),input^Y(M_2)\right] & \hbox{otherwise.}  \end{array}\right. $$

*Y* runs on the intermediate values computed by *X*, producing nonsense, until *X* reaches its halting state (indicated by 0 = *value*_13_). Finally, working from *X*’s halting state, *Y*’s calculations break away and continue on their own. For *Y*, there may be many false starts, but the values and inputs from these false starts are forgotten each time RaiN signals (by 0 < *value*_13_) a change in *X*. Communication from *XH*? to *Y* is seen in the first lines of each definition. If *X* becomes trapped in an attractor without ever halting, then *Y* continues to generate nonsense. So the composition succeeds only if *X* halts and then *Y* halts working from the state left by *X*.

## TearS of the Sun

The author worked with Dr Leon Kotin (Fort Monmouth, NJ) in the 1970s and 1980s to develop early versions of this process—see (Stark and Kotin 1987) for an informal presentation. In the past two or three decades many others e.g., Hubbell and Han (2012) have further developed amorphous sensor networks.

A network with a moving cell *r* is represented as (*C*_*n*_, *E*_*n*_) where *E*_*n*_(*c*) − {*r*} is more-or-less constant for *c* ≠ *r* and *E*_*n*_(*r*) is variable, $n=0,1,\ldots$ (Stark and Kotin 1987). With *r* as its root and *N* large, GradienT_*N*_ is constantly re-computing the cell-to-root distances. A global input $x_n:C_n\rightarrow\{0,1,\ldots\}$ of sensor reports—*x*_*n*_(*c*) = 0 representing no alarm—is maintained at the cells. Occasionally, the movement of unknown individuals (i.e., biological, infra-red emitters) past a cell *c* is detected and expressed as 0 < *x*_*n*_(*c*). These sensing cells are probably stationary. The objects being detected are not a part of the network. A mechanism for moving information, describing the location (relative to *r*) of alarms, down gradient to *r* is included. I call this canopy intelligence process **TearS** (a variation on RaiN). The information consists of *value*_1_ the nature of the alarm and *value*_3_ the distance to the nearest alarmed cell.

Let $Q=\{0,1\ldots\}\times\{0,1,\ldots,N\}\times\{0,1,\ldots,N\},$ where *N* is at least the diameter of the network. The structure of $s(c)=value\in Q$ is broken down into *s*(*c*)_1_ = *x*_*n*_(*c*) sensor input at *c*, *s*(*c*)_2_ is computed by GradienT_*N*_ to be *c*’s distance from *r*, and *s*(*c*)_3_ the distance from $s^{-1}(\{1,\ldots\})$—the set of alarmed cells. TearS is defined by

$$ \alpha(value,input) = \left\{\begin{array}{ll}   \left[value_1,\ 0,\ (input_3+1)\right]       & \hbox{for } c=r, \\ \left[value_1,\ (input_2+1),\ 0\right]       & \hbox{for } 0<value_1, \\   \left[value_1,\ (input_2+1),\ (input_3+1)\right] & \hbox{otherwise,}  \end{array}\right. $$

and for $$M\subset Q$$

$$ input(M) = [max(M_1),min(M_2),min(M_3)]. $$

The root is not necessarily the only changing part of the network. Cells die and randomly-positioned replacements are rewoven into (*C*_*n*_, *E*_*n*_)—using this simple program. With a little more work the information passed to the root could include the threat’s size and structure e.g., Han (2012).

TearS processes a dynamic stream of data *x*_*n*_ in constantly changing network (*C*_*n*_, *E*_*n*_). The name was inspired by a similar process depicted in the movie Tears of the Sun (Fuqua 2003). Half-way through the movie, thousands of solar-powered infra-red sensors, *C* − {*r*}, are dropped into the canopy of a forest. Using radio-frequency packet-passing, they begin building a network *E*_*n*_. A lap-top *r* carried by the hero is included in the network. The environment takes its toll, requiring new sensors to be dropped in as old sensors retire—(*C*_*n*+1_,*E*_*n*+1_). The network re-weaves itself so that the hero is always informed of the position of the advancing bad guys.

## Conclusion

$\ldots$ The greatest challenge today in all of science, is the accurate and complete description of complex systems. Scientists have broken down many kinds of systems. The next task is to reassemble them, at least in mathematical models that capture the key properties of the entire ensembles. (Wilson 1998)

My hope is to see the development of a powerful computation theory for issues of biological information processing. The absolutely amorphous model is mathematically tractable and approximates both mature biological tissues and the informal notion of amorphous computing seen in current literature. This brings insights obtained through pure mathematics close enough to reality to be biologically relevant—just as Turing’s leopards’ spots problem (Turing 1952) and its solution highlighted the value of reaction-diffusion mechanisms in theoretical biology.

It is clear that the methods which I called blind search is not biologically realistic—a leopard embryo cannot invest geological time into computing its spots. This demonstrates the importance of gradients on (*Q* ^*C*^, ⇒) and activity organized into waves as a means of directing development. The entropy gradient championed by Schrödingier (1944) may be the most promising, since Shannon entropy (Shannon 1998) equates morphogenesis to information-reduction. These issues are a part of computational thermodynamics (Bennett 2003; Feynman 1996).

Non-homogeneous processes are central to biology. Perhaps models developed in this formalism can suggest a need for non-homogeneity in toy organs. 22

Classical computation theory can offer guidance, 23 but is the powerful integer-coding used classically impossible or inappropriate 24 for amorphous computation?

I cannot believe that amorphous computing is, as some have speculated, completed. Three billion years of evolution have left us with fantastic processes which need mathematical modeling.

## Footnotes

1 Since Floyd (1967a, b), and continuing with Broy (1986), Ward and Hayes (1991), and others, attention had been focused on individual runs/computations. Infinitely often these runs are successful (a result of *angelic* decisions) and infinitely often they fail (a result of *demonic* decisions). This approach to understanding non-deterministic processes is as inappropriate as viewing real numbers one decimal at a time. For arithmetic, viewing decimals in this way might have made the existence of irrationals a subject of debate. For processes we see something similar in Dijkstra (1988) and Schneider and Lamport (1988).

2 My assumption that activity is random has mathematical advantages (see the proof of Theorem 3), but it allows extreme differences between the activity levels of healthy cells—differences that would not be seen Nature.

3 Cell-to-cell edges may correspond to gap-junctions in animal tissue. Gap junctions are non-directed channels between the cytoplasms of neighboring cells which allow the exchange of small molecules (up to mw = 2,000). Viruses are too large to pass through gap junctions, so if our model is to be biologically true, program fragments should not pass between cells. This excludes many tricks seen in recursion theory. Besides gap-junctions, cell-to-cell communication may be hormonal (broadcast), mechanical, and electrical. Physically independent bacteria communicate. In fact amorphous information sharing between members of a species is ubiquitous in Nature.

4 A graph/network (*C*, *E*) is *bipartite* if *C* can be partitioned into *C*_0_, *C*_1_ so that every edge *xy* of *E* connects a cell in *C*_0_ to a cell in *C*_1_. *C*_0_ = *s* ^−1^(0) and *C*_1_ = *s* ^−1^(1) for a halting *s*. Being bipartite is a global property—if true, it cannot be determined by examining less than the whole graph/network.

5 This theorem does not say that the expected halting time is *N* ≤ ||*C*||.

6 “von Neumann thought that automata mathematics *should be closer to the continuous and should draw heavily on analysis* as opposed to [the] combinatorial approach” (Burkes 1970). Theorem 3 is an example of a 0,1-law.

7 For the analytical approach, think of $(Q^C)^\mathbb{N}$ as being analogous to the unit interval [0, 1]⊂ ℜ, and computations corresponding to decimals in $\{0,1,\ldots 9\}^\mathbb{N}$. Consider the partial function $h:(Q^C)^\mathbb{N}\rightarrow \mathbb{N}$ which, for each ${\overline s}=\langle s_0,\ldots s_i,\ldots,\rangle,$ is defined

$$ h({\overline s}) = \left\{\begin{array}{ll}\min i\ [s_i is \; a \; halting \; state ] & \hbox{if}\;{\overline s}\; \hbox{is a halting computation}, \\ undefined & \hbox{otherwise.} \end{array}\right. $$

For a process which halts with probability 1, the expected halting time is

$$ \int\limits_{Q^{C}} h({\overline s}) d \mu({\overline s}). $$

This would be the sort of analytical result that von Neumann had in mind.

8 Alternate 0,1 value assignments as you move around *Cm*, until the last cell which is set to 2.

9 Any three cells with values 0,1,2 form a triangle, so the fourth cell cannot be stable with one of these values.

10 The input *s*_0_ consists of *x* = ||*C*|| · log_2_(||*Q*||) bits of information, so *P* and *NP* would be sets of computations of length *p*(*x*), where *p* is a polynomial.

11 I conjecture that, if the non-determinism seen in *σ*-selection is pseudo-random, rather than strictly random, then there can exist a tripartite graph and *s*_0_ for which 3PartitioN fails. The failure would of course be due to the computed $\ldots\sigma_m,\ldots$ containing a pattern which perpetuated instability. This could also be true of halting for 2PartitioN. To give the reader something to work with, I assume that *pseudo-random* means computable using memory which is linear in ||*C*|| + ||*Q*||—as if the process were being simulated in a machine which generates the $\ldots\sigma_m,\ldots$ with this bound on memory.

12 “Non-homogeneous” because one cell is not executing the *α* used by the other cells. Not absolutely amorphous because the designation of an inactive cell takes place outside of *α*, *input*. The gradient sensitivity of 2PartitioN* is, on the other hand, defined within *input* and based only on local information.

13 Imagine *s*4 ⇒_*σ*_ *s*5. In 2PartitioN, *σ* = {1, 2, 3} is only possibility, so *T*_5,4_ = 2^−6^ = 16 · 2^−10^. But in 2PartitioN* a second possibility is *σ* = {1, 2, 3, 4}, and in each cell has a 2^−1^ chance of receiving the appropriate input, so the probability of the transition is $T_{5,4}^{\ast}$ = 2^−6^2^−3^ + 2^−6^2^−4^ = 3 · 2^−10^. For the transition *s*6 ⇒_*σ*_ *s*6, eight cases make *T*_6,6_ = 2^−3^ and 32 cases make $T_{6,6}^{\ast}$ = 9 · 2^−5^ > 2^−2^.

14 From *s* = 0131 on *C*4 we have *B*_0131_ = 2. There will be three, out of 16 possible next-states, which move up-gradient—*B*_0242_ = 4, *B*_0241_ = 3, *B*_0142_ = 3.

15 See videos of tissues it at http://www.youtube.com/watch?v=Y5uKMM8Od9g or http://www.youtube.com/watch?v=BJTFeBGO_i0 or Google *“cardiomyocytes beating in vitro”*.

16 Indexed over *C*, *E*_*c*,*d*_ = 1 if *cd* is an edge, =0 otherwise.

17 Dereniowski and Pelc (2012) capture dynamics by allowing identical anonymous agents to move through a given fixed graph. They answer the question *“for which initial configurations of agents is leader election possible"*. Agents which are Turing machines which are initially given an upper bound on the size of the graph. They show *“leader election is possible when the initial configuration is asymetric and agents can learn [the asymetry], regardless of the actions of the adversary [demon]"*, so their result is not inconsistent with the non-computability result proved here.

18 Given a measure μ on set of events, we say that an action *almost always* succeeds if and only if the set of successes has measure 1, and the set of failures has measure 0.

19
$F\subseteq E$ is a cut-set for the connected graph (*C*, *E*) if (*C*, *E* − *F*) is not connected

20 In a 1980 paper, Angluin (1980) proved *there exists no election algorithm for a class of graphs containing both a graph* *G* *and a strict covering* *G*′ *of* *G* *[by* γ*]*. Her proof (not unlike this proof) carried computation steps on edges *ab* of *G* back to γ^−1^(*ab*) of *G*′ to reach a contradiction [on *G*′].

21 Let ${\mathbb{N}^*}$ be the least set containing ${\mathbb{N}}$ and closed under the formation of lists. Most of the model described in Sect. 2 can be developed in ${\mathbb{N}^*}$—i.e., *C* = [0, 1, …*p*] is a set of cells, $E\subseteq C^2$ is a list of pairs, *Q* = [0, 1, …*q*], *Q* ^+^ = [0, 1, … *r*] for *q* ≤ *r* and $\alpha\subset Q\times Q^+\times Q$ is a list of ordered triples. Multisets are represented as counting vectors indexed over *Q*—i.e., [0, 3, 0, 0, 1, 0] represents {1, 1, 1, 4} when *q* = 5. We have put no upper bound on the degree of cells, so *Input* has an infinite domain and so it must remain a defined but computable recursive function. Only the random choice function is excluded, but our processes’ deterministic state-to-halting-state functions can be defined random choice. Details are available in Stark (2013).

22 As a toy organ, consider three homogeneous processes—*α* on *X*, *β* on *Y* and γ on *Z*—with (*C*, *E*) partitioned by connected subgraphs  and  separated by the cut set  . Can we design a computational need for *Y*? Perhaps state-changes over *X* are filtered by *Y* so that *Z* sees only unusual events within *X*. Could something like this lead to a theoretical justification for the evolution of organs?

23 For example, like automata, the amorphous model has limited memory, but does the elegant proof that the palindromes of a given language cannot be recognized by an automaton carry over to amorphous processes? Or, could it be that the *C*_1_ *C*_2_ construction used as a counterexample to ElectioN, is a version of the construction of a palindrome failure?

24 (*C*, *E*) offers some coding ability, but using it may be inconsistent with the amorphous philosophy.
